# Germline DNA Repair Gene Mutations in Young-onset Prostate Cancer Cases in the UK: Evidence for a More Extensive Genetic Panel

**DOI:** 10.1016/j.eururo.2019.01.050

**Published:** 2019-09

**Authors:** Daniel A. Leongamornlert, Edward J. Saunders, Sarah Wakerell, Ian Whitmore, Tokhir Dadaev, Clara Cieza-Borrella, Sarah Benafif, Mark N. Brook, Jenny L. Donovan, Freddie C. Hamdy, David E. Neal, Kenneth Muir, Koveela Govindasami, David V. Conti, Zsofia Kote-Jarai, Rosalind A. Eeles

**Affiliations:** a Oncogenetics, Division of Genetics and Epidemiology, The Institute of Cancer Research, London, UK; b School of Social and Community Medicine, University of Bristol, Bristol, UK; c Nuffield Department of Surgical Sciences, University of Oxford, Oxford, UK; d Faculty of Medical Science, John Radcliffe Hospital, University of Oxford, Oxford, UK; e Department of Oncology, Addenbrooke's Hospital, University of Cambridge, Cambridge, UK; f Cancer Research UK Cambridge Research Institute, Li Ka Shing Centre, Cambridge, UK; g Division of Population Health, University of Manchester, Manchester, UK; h Department of Preventive Medicine, Keck School of Medicine, University of Southern California/Norris Comprehensive Cancer Center, Los Angeles, CA, USA; i The Royal Marsden NHS Foundation Trust, London, UK

**Keywords:** Prostate cancer, DNA repair genes, Genetic predisposition, Gene panel testing, Aggressive phenotype

## Abstract

**Background:**

Rare germline mutations in DNA repair genes are associated with prostate cancer (PCa) predisposition and prognosis.

**Objective:**

To quantify the frequency of germline DNA repair gene mutations in UK PCa cases and controls, in order to more comprehensively evaluate the contribution of individual genes to overall PCa risk and likelihood of aggressive disease.

**Design, setting, and participants:**

We sequenced 167 DNA repair and eight PCa candidate genes in a UK-based cohort of 1281 young-onset PCa cases (diagnosed at ≤60 yr) and 1160 selected controls.

**Outcome measurements and statistical analysis:**

Gene-level SKAT-O and gene-set adaptive combination of *p* values (ADA) analyses were performed separately for cases versus controls, and aggressive (Gleason score ≥8, *n* = 201) versus nonaggressive (Gleason score ≤7, *n* = 1048) cases.

**Results and limitations:**

We identified 233 unique protein truncating variants (PTVs) with minor allele frequency <0.5% in controls in 97 genes. The total proportion of PTV carriers was higher in cases than in controls (15% vs 12%, odds ratio [OR] = 1.29, 95% confidence interval [CI] 1.01–1.64, *p* = 0.036). Gene-level analyses selected *NBN* (*p*_*SKAT-O*_ = 2.4 × 10^−4^) for overall risk and *XPC* (*p*_*SKAT-O*_ = 1.6 × 10^−4^) for aggressive disease, both at candidate-level significance (*p* < 3.1 × 10^−4^ and *p* < 3.4 × 10^−4^, respectively). Gene-set analysis identified a subset of 20 genes associated with increased PCa risk (OR = 3.2, 95% CI 2.1–4.8, *p*_*ADA*_ = 4.1 × 10^−3^) and four genes that increased risk of aggressive disease (OR = 11.2, 95% CI 4.6–27.7, *p*_*ADA*_ = 5.6 × 10^−3^), three of which overlap the predisposition gene set.

**Conclusions:**

The union of the gene-level and gene-set-level analyses identified 23 unique DNA repair genes associated with PCa predisposition or risk of aggressive disease. These findings will help facilitate the development of a PCa-specific sequencing panel with both predictive and prognostic potential.

**Patient summary:**

This large sequencing study assessed the rate of inherited DNA repair gene mutations between prostate cancer patients and disease-free men. A panel of 23 genes was identified, which may improve risk prediction or treatment pathways in future clinical practice.

## Take Home Message

This large sequencing study assessed the rate of inherited DNA repair gene mutations in prostate cancer (PCa) patients and in disease-free men. A total of 23 genes were found to be associated with PCa predisposition or the risk of aggressive disease. These findings have predictive and prognostic potential.

## 1 Introduction

Prostate cancer (PCa) is the most common solid tumour in men living in the developed world besides nonmelanoma skin cancer and responsible for over 300 000 deaths per year worldwide [1], although the majority of PCa cases are diagnosed with low- or intermediate-risk disease. Family history (FH) is a strong risk factor for PCa, and twin studies demonstrate a large contribution by heritable genetic factors [2]. Increasing evidence indicates that both common and rare germline variation contribute to PCa predisposition [3], [4]. Rare loss of function (LoF) germline mutations in *BRCA2* have convincingly been implicated as contributing to both FH of PCa and increased likelihood of aggressive disease with poor prognosis, whilst lower mutational frequencies or less consistent evidence has also been presented for a small subset of additional DNA repair genes including *ATM*, *BRCA1*, *BRIP1*, *CHEK2*, *GEN1*, *MSH2*, *NBN*, *PALB2* and *RAD51D* [5], [6], [7].

In this study, we performed screening of 167 genes from DNA damage response and repair pathways within a large UK-based case–control cohort with long follow-up, to further investigate the role of germline DNA repair gene mutations in PCa predisposition, clinical outcome, and survival. To maximise the power in this study, we utilised young-onset cases (diagnosed at ≤60 yr) and control samples screened for either no PCa FH or low prostate-specific antigen (PSA; <0.5 ng/ml). These results should help inform the composition of future gene panels for clinical screening and risk profiling.

## 2 Patients and methods

### 2.1 Study population

Self-reported European ancestry PCa cases were randomly selected from the young-onset (diagnosed at ≤60 yr) subcohort of the UK Genetic Prostate Cancer Study (UKGPCS) [8]. Control men with no FH of PCa were recruited from GP practices participating in UKGPCS, or those with PSA <0.5 ng/ml were recruited from the Prostate Testing for Cancer and Treatment (ProtecT) trial [9]. Cases and controls were matched for genetic ancestry, with ethnicity confirmed for all samples by principal component analysis and analyses restricted to genetically European ancestry individuals (Supplementary material, Methods, and Supplementary Figs. 1 and 2). No formal matching by age was performed, although the age profiles of the case cohort and control men with known age at recruitment were broadly similar (Table 1). All studies were approved by the appropriate ethics committees (UKGPCS 848). All participants gave written informed consent.

**Table 1:** Summary of study cohort characteristics

Clinical variable	Cases (*n* = 1281)	Controls (*n* = 1160)
Age of diagnosis (cases) or blood draw (controls)		
Median	57	56
Quartiles	54–58	53–59
Range	38–60	44–67
Unknown (count)	0 (0%)	637 (55%)
Ethnicity		
European ancestry	1281 (100%)	1160 (100%)
Diagnosis method		
Clinical symptoms	739 (58%)	–
Screen detected	403 (31%)	–
Unknown	139 (11%)	–
PCa family history		
0	973 (76%)	510 (44%)
1	207 (16%)	17 (1.5%)
2	40 (3.1%))	1 (0.1%)
3+	5 (0.4%)	–
Unknown	56 (4.4%)	632 (54%)
PSA at diagnosis (ng/ml)		
Median	8.4	–
Quartiles	5.6–18.3	–
Range	0.04–9020	–
Unknown (count)	43 (3.4%)	–
Gleason score (highest recorded)		
≤6	576	–
7	472	–
≥8	201	–
Unknown	32	–
Primary tumour stage at diagnosis		
T1	365 (28%)	–
T2	524 (41%)	–
T3	295 (23%)	–
T4	63 (4.9%)	–
T_*X*_	34 (2.7%)	–
Lymph node status at diagnosis		
N0	787 (61%)	–
N1	89 (6.9%)	–
N_*X*_	405 (32%)	–
Distant metastases at diagnosis		
M0	757 (59%)	–
M1	92 (7.2%)	–
M_*X*_	432 (34%)	–

PCa = prostate cancer; PSA = prostate-specific antigen.

Analyses were performed comparing all post-quality control (QC) PCa cases (*n* = 1281) versus controls (*n* = 1160), and for case–case comparisons of aggressive (Gleason score ≥8, *n* = 201) versus nonaggressive (Gleason score ≤7, *n* = 1048) cases (Table 1).

### 2.2 Target genes

We constructed a 175 gene sequencing panel after a literature review of DNA repair, damage response and cell cycle pathways, and databases (Supplementary material, Methods). The panel comprised 107 genes in DNA repair pathways, 60 DNA damage response and cell cycle regulation genes, and eight other candidate PCa predisposition genes (*HOXB13, MSR1, RNASEL*, *AR, ESR1, ESR2*, *NKX3-1*, and *SPOP*; Table 2 and Supplementary Table 1).

**Table 2:** Summary of gene panel composition by primary DNA repair pathway

Consensus pathway	Total number of genes
Direct reversal repair (DRR)	3
Base excision repair (BER)	25
Mismatch repair (MMR)	12
Nucleotide excision repair (NER)	30
Homologous recombination (HR)	26
Nonhomologous end joining (NHEJ)	11
Fanconi anaemia (FA)	19
DNA damage response (DDR)	22
Cell cycle regulation	19
PCa candidates	8

**Total**	**175**

PCa = prostate cancer.

### 2.3 Target capture and sequencing

A custom SureSelect XT bait library (Agilent Technologies, Santa Clara, CA, USA) was designed for coding regions of the 175 target genes. DNA libraries were prepared using an automated in-house sample preparation protocol (Supplementary material, Methods) and captured libraries sequenced using Illumina HiSeq 2000 v4 chemistry (Illumina, San Diego, CA, USA).

### 2.4 Sequence data analysis, variant annotation, and QC

Raw sequencing reads were aligned to GRCh37 using BWA 0.5.8 [10]. Samples reaching ≥80% of the target at ≥10× read depth as defined by Picard v.1.52 (http://broadinstitute.github.io/picard/) and contamination <3% as estimated by verifyBamID v1.1.1 (https://github.com/statgen/verifyBamID/releases) were genotyped using GATK v2.8-1 [11]. Per-gene coverage levels were assessed using the GATK tool “DiagnoseTargets”, with a per-base coverage QC threshold set at ≥8 reads at base quality ≥20. Low-quality genotypes were removed according to established thresholds (Supplementary material, Methods) [12], [13], [14]. Standard QC procedures were applied to remove poorly performing samples and variants [15]. These include variant-level filters such as heterozygosity and missingness (Supplementary Fig. 3), and sample-level filters including relatedness and divergent ancestry (Supplementary material, Methods). Owing to the targeted nature of the sequencing data, ancestry QC was augmented with additional QC data from the OncoArray platform [16].

Variants were annotated by wANNOVAR [17] using RefSeq Gene definitions [18], and variant consequence was checked using Variant Effect Predictor (VEP; release 84, March 2016) [19]. Protein truncating variants (PTVs; frameshift Indels, stop gain, and splice variants) were also annotated with the VEP plugin Loss-of-Function Transcript Effect Estimator (LOFTEE; https://github.com/konradjk/loftee/), and Indels in splice sites were manually reviewed for consequence. For further analysis, variants were categorised into two groups: (1) tier 1 contained all high-confidence PTVs according to LOFTEE and manual splice-site review and (2) tier 2 contained all remaining variants with Combined Annotation Dependent Depletion (CADD) v1.3 score >20 [20].

### 2.5 PCa susceptibility gene identification

Comparisons of rare PTV frequencies between our cohort and previous publications were restricted to tier 1 mutations with minor allele frequency (MAF) <0.5% in our controls. For novel gene discovery tests, due to the low frequencies of individual variants in this study, we performed two distinct aggregate statistical tests for each study phenotype: (1) a gene-level SNP-set association test over all genes containing two or more tier 1 or 2 variants and (2) a gene-set-level association test where tier 1 mutations with MAF <0.5% in controls were collapsed per gene.

To identify associated genes (test 1) we used SKAT-O, a unified test able to tolerate the inclusion of neutral variants or variants with opposing direction of effect, which finds the optimal combination between burden and kernel tests for the tested data [21]. SKAT-O was run unadjusted over genes containing two or more variants (tier 1 or tier 2), with statistical significance set at a Bonferroni adjusted *p* value of *α* = 0.05/number of genes; *p *< 3.1 × 10^−4^ for case/control analysis (159 genes), and *p *< 3.4 × 10^−4^ for aggressive phenotype analysis (146 genes). To further investigate gene-level SKAT-O association signals, we used adaptive combination of *p* values (ADA), a “combination of *p* values” method that adaptively truncates *p* values with an optimal threshold for the tested data set, removing neutral variants and identifying the likely underlying variant-level components of the gene-level signal [22]. Gene-level ADA for genes identified by SKAT-O was run using all tier 1 and 2 variants within these genes and default settings (corresponding to *p* value truncation thresholds of 0.1–0.2 considered in 0.01 increments) except for increasing to 10 000 permutations and using the mid *p* value setting [23].

We subsequently performed an additional gene discovery analysis (test 2) in which ADA was used to identify a candidate gene set rather than individual variants, by collapsing tier 1 mutations with MAF <0.5% in controls on a per-gene basis rather than a variant-level basis (except for *CHEK2* where 1100delC was a separated from all other *CHEK2* PTVs due to its relatively higher frequency), under the assumption that rare tier 1 variants are more likely to confer a homogenous effect within each gene. For each phenotype, gene-set-level ADA was run with default settings except for mode = “dominant”, twoSided = F, midp = TRUE, and 10 000 permutations. We report both the permuted *p* value (*P*_*ADA*_) and the truncation threshold (opt.t). To display the resulting gene set selected by ADA, forest plots were constructed showing gene-level adjusted odds ratios (ORs) calculated from the collapsed tier 1 MAF <0.5% variant count using unadjusted Firth's regression.

### 2.6 Survival analysis

Survival analyses were performed within the PCa case cohort to examine the effect of gene set's carrier status on patient outcome. The follow-up period was based on the date of diagnosis, date of consent into the UKGPCS, and date of last follow-up. Cases were diagnosed and came under observation at the date of consent. Survival time was calculated as the difference in time between age of diagnosis and the last recorded follow-up or date of death.

Kaplan-Meier survival analysis and univariable Cox regression models, adjusted for age, were performed. Log-rank tests were performed to investigate the equality of survivor functions across gene sets. Multivariable Cox regression models of gene set carrier status were constructed, adjusted for age and all covariates significant at *p *< 0.05 under Cox univariate regression. All survival analyses were performed in Stata 14.2 [24].

## 3 Results

### 3.1 Sequencing and sample summary

After QC, variant data were available for 1281 PCa cases and 1160 control samples. Of 175 genes targeted, three (*GTF2H2*, *SLX1A*, and *SLX1B*) were excluded due to low coverage resulting from segmental duplication and one (*PRKDC*) was removed as wANNOVAR was unable to annotate coding consequences due to an incomplete RefSeq gene definition (Supplementary Fig. 4 and Supplementary Table 2). From the 171 tractable target genes, we classified 2078 variants in 164 genes as tier 1 or 2 (Supplementary Table 3).

### 3.2 Known gene-set enrichment

A total of 233 PTVs with MAF <0.5% in controls were identified in 97 of the genes passing QC. Overall PTV carrier burden was significantly enriched in PCa cases compared with controls (15% vs 12%; *p* = 0.036). This enrichment was greater within the BROCA panel of cancer predisposition genes, primarily focussed on hormone-driven breast and ovarian cancers (http://web.labmed.washington.edu/tests/genetics/BROCA_VERSIONS) [25]. For the original 22 gene BROCA panel, 57 PTVs were identified in 15 genes (4.5% in cases vs 2.2% in controls; *p* = 2.5 × 10^−3^), whilst for the current BROCA-v7 containing 66 genes, 80 PTVs were identified in 23 genes (5.5% in cases vs 3.5% in controls; *p* = 0.020). The greatest enrichment was for the top five genes reported by Pritchard et al. [7] (*ATM*, *BRCA1*, *BRCA2*, *CHEK2*, and *GEN1*), with 38 total PTVs identified across all five genes (3.8% vs 1.4%; *p* = 2.1 × 10^−4^).

### 3.3 Gene-level association

**Fig. 1:**
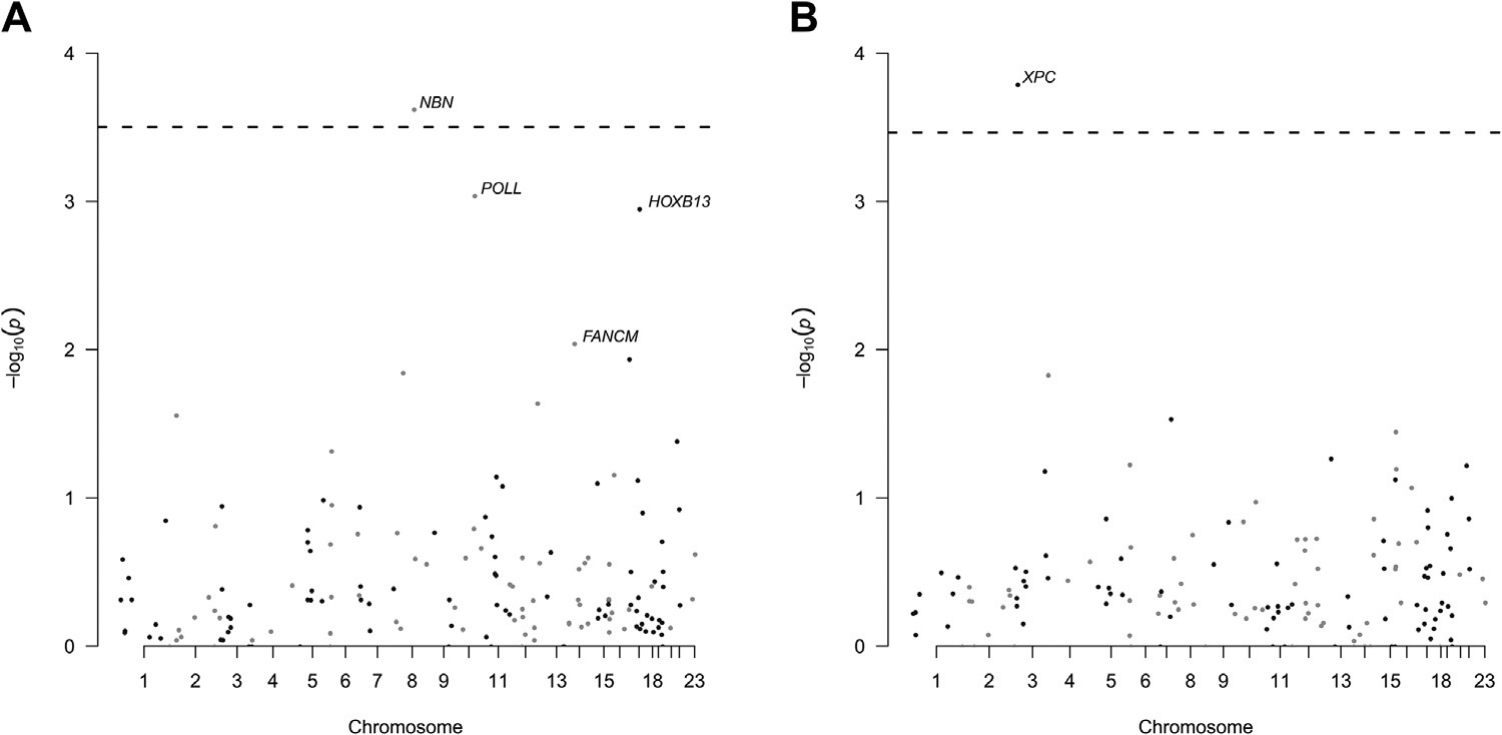
SKAT-O results for (A) case–control and (B) aggressive phenotypes. The dashed line denotes the Bonferroni-corrected candidate-level significance threshold for each phenotype, according to the number of genes containing two or more tier 1 and 2 PTVs included in the analysis (159 and 146 respectively). Genes are labelled at *p* < 0.05. PTV = protein truncating variant.

Gene-level analyses were restricted to genes containing two or more tier 1 and 2 variants. In the case/control analysis (159 genes tested) *NBN* reached significance (*p* = 2.4 × 10^−4^; *p* = 0.18 for aggressiveness), as did *XPC* for the aggressive phenotype (146 genes tested; *p* = 1.6 × 10^−4^, *p *= 0.90 for overall PCa; Fig. 1, and Supplementary Figs. 5 and 6). In addition, *HOXB13* (*p* = 1.1 × 10^−3^; *p* = 0.12 for aggressiveness) and *POLL* (*p* = 9.1 × 10^−4^; *p* = 0.11 for aggressiveness) demonstrated nominal significance (*p *< 0.05) in the case/control analysis.

To further investigate these SKAT-O association signals, we used ADA to interrogate the combination of variants contributing to the association (*HOXB13* and *POLL* were also included due to the well-characterised role of *HOXB13* in PCa predisposition). For both *NBN* and *HOXB13*, ADA identified a single-recurrent heterozygous nonsynonymous variant enriched among PCa cases to be responsible for the gene-level signal, whilst for *POLL,* four of the 15 tested variants were identified to be responsible for potentially modulating risk (three protective and one pathogenic). For *XPC*, ADA selected six singleton heterozygous variants from the nine variants tested as contributing to the aggressive phenotype, all of which were observed in different individuals (Table 3).

**Table 3:** Variant-level investigation of genes nominally significant in the SKAT-O gene-level analysis of tier 1 and 2 variants

Gene (variants tested)	ADA-selected variants	rsID	Tier	Case (*n* = 1281)	Control (*n* = 1160)	CADD	ExAC NFE	Variant *p* value
**Case–control phenotype**								
*NBN* (4)	8:90993640_C/T	rs61753720	2	18	2	26.3	0.0030	4.3 × 10^−4^
*POLL* (15)	10:103339221_G/A	rs555309980	2	3	0	34	0.000047	0.13
	10:103339487_C/T	rs200705693	2	0	2	22.3	0.000091	0.20
	10:103342648_C/T	rs139871590	2	1	5	34	0.0015	0.09
	10:103343423_G/A	rs142726673	2	0	10	23.7	0.00080	4.7 × 10^−4^
*HOXB13* (9)	17:46805705_C/T	rs138213197	2	20	3	29.6	0.0031	5.9 × 10^−4^
**Aggressive phenotype**								
*XPC* (9)	3:14187577_G/A	–	2	1	0	23.5	0.000015	0.07
	3:14193884_G/A	rs3731152	2	1	0	31	0.000033	0.07
	3:14199634_C/G	–	2	1	0	26.8	–	0.07
	3:14208716_T/C	rs200485886	2	1	0	24.7	0.000078	0.07
	3:14209787_G/A	rs188716339	2	1	0	24.2	0.000031	0.07
	3:14214457_G/A	–	2	1	0	22.8	–	0.07

ADA = adaptive combination of *p* values; NFE = non-Finnish Europeans.

The number of unique variants per gene tested, individual variants selected by ADA, case and control variant counts, variant CADD v1.3 score, minor allele frequency in ExAC NFEs, and variant-level *p* values (using unadjusted Firth's logistic regression) are shown for each variant selected by ADA.

### 3.4 Candidate gene-set discovery

**Fig. 2:**
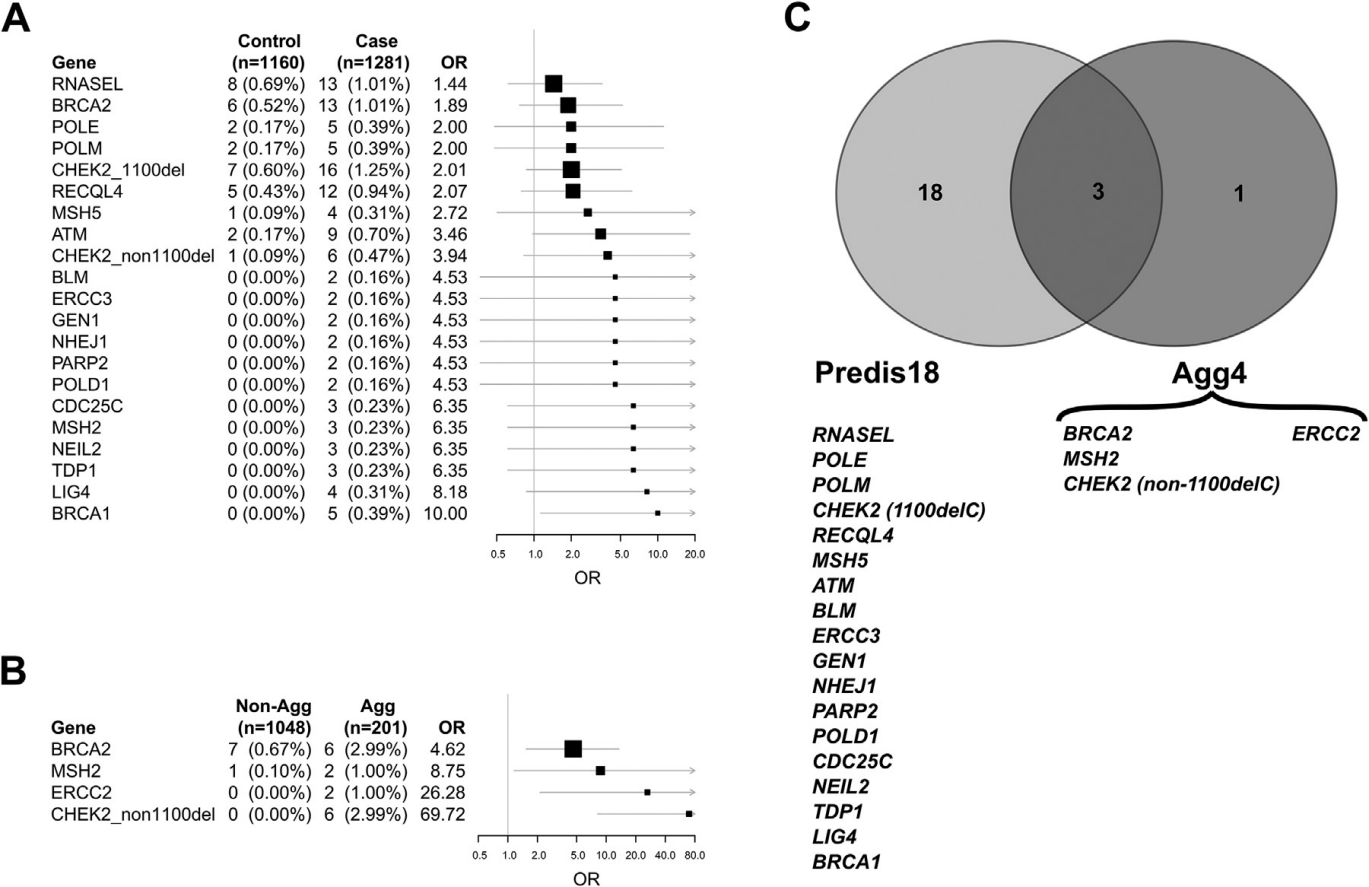
Gene set selection. Forest plots of (A) 20 unique genes selected by ADA case–control analysis and (B) four genes selected by ADA aggressive phenotype analysis. Odds ratios (ORs) were estimated from the collapsed tier 1 MAF <0.5% variant count using unadjusted Firth's logistic regression, with 0.5 added to each count to provide estimates for genes with no carriers in one cohort. (C) Intersection of gene sets from the case-control and aggressiveness analyses and partition into non overlapping Predis18 and Agg4 gene panels. ADA = adaptive combination of *p* values; MAF = minor allele frequency.

For the case/control phenotype, ADA selected 20 distinct genes containing rare heterozygous PTVs from a panel of 57 genes (both categories of *CHEK2* PTV selected). These genes were significantly enriched among PCa cases compared with controls (8.5% vs 2.8%, OR = 3.2, 95% confidence interval [CI] 2.1–4.8, *p*_*ADA*_ = 4.1 × 10^−3^, opt.t = 0.2; Fig. 2A), and eight patients were carriers of more than one PTV (Supplementary Table 4). Only five of these genes (*ATM*, *BRCA1*, *BRCA2*, *CHEK2*, and *MSH2*) overlap the BROCA 22 gene set. In the aggressive phenotype analysis, out of 35 genes, ADA selected four that were significantly enriched in Gleason ≥8 cases in comparison with Gleason ≤7 patients (8.0% vs 0.8%, OR = 11.2, 95% CI 4.6–27.7, *p*_*ADA*_ = 5.6 × 10^−3^, opt.t = 0.1; Fig. 2B). Three of these genes overlap with the case/control gene set (*BRCA2*, *CHEK2*, and *MSH2*), whilst *ERCC2* is unique to the aggressive set. In contrast to other *CHEK2* PTVs, the *CHEK2* 1100delC variant was not enriched among aggressive cases.

**Fig. 3:**
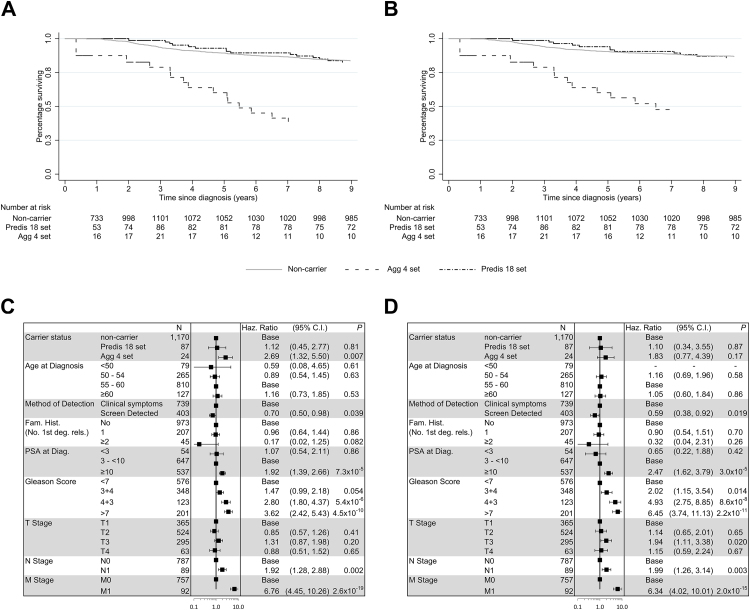
Gene set survival. Kaplan-Meier survival plots depicting (A) overall survival and (B) cause-specific survival. Multivariate Cox regression analysis of phenotypic features and gene set carrier status are shown for (C) overall survival and (D) cause-specific survival. Analyses were conducted using PCa cases only. CI = confidence interval; diag. = diagnosis; Fam. hist. = family history; Haz. ratio = hazard ratio; PCa = prostate cancer; PSA = prostate-specific antigen; 1st deg. rels. = first-degree relatives.

The combined set of 21 genes identified in these analyses demonstrated a continuum of aggressive phenotype risk (Supplementary Fig. 7), with the upper tail defining predisposition genes with a lower risk of aggressive disease and the lower tail the converse. We partitioned the gene set into nonoverlapping sets of 18 genes in the predisposition panel (Predis18) and four in the aggressive panel (Agg4), with *CHEK2* split (1100delC in Predis18 and other PTVs in Agg4; Fig. 2C). As would be expected, given the phenotype criteria, Agg4 carriers showed significant enrichment for several clinical indicators of aggressive disease (higher PSA, Gleason score, tumour stage, and nodal spread). Predis18 carriers showed no association with any clinical variable (Table 4). A modest increase in PCa FH rate was observed among Predis18 carriers compared with noncarriers, whilst PCa FH rates were lower among Agg4 carriers; however, both these trends were nonsignificant. Suggestive but nonsignificant increases in rates of breast and pancreatic cancer FH were also observed for carriers of the Agg4 gene set (Supplementary Table 5). Kaplan-Meier survival analysis showed a significant global difference across gene-set carriers (Agg4, Predis18, and noncarriers) for both all-cause and PCa-specific mortality (log-rank test, *p*_all-cause_ = 9.8 × 10^−8^, *p*_PCa-specific_ = 4.1 × 10^−6^). This is attributable to Agg4 carriers demonstrating significantly worse survival than noncarriers, as survival between Predis18 carriers and noncarriers was very similar. For all-cause survival (Fig. 3A), 5-yr survival rates were 60% for Agg4 (95% CI 34–79%), 93% for Predis18 (95% CI 85–97%), and 89% for noncarriers (95% CI 87–91%). The hazard ratio for Agg4 carriers compared with noncarriers was 2.69 (95% CI 1.32–5.50; Fig. 3C). A similar pattern was observed when considering only PCa-specific survival (Fig. 3B), though hazard ratios were not statistically significant, possibly due to the reduction in the number of events (282 compared with 212). Five-year survival rates were 60% for Agg4 (95% CI 34–79%), 94% for Predis18 (95% CI 86–98%), and 91% for noncarriers (95% CI 89–92%). The hazard ratio for Agg4 carriers compared with noncarriers was 1.83 (95% CI 0.77–4.39; Fig. 3D).

**Table 4:** Clinical characteristics of Predis18 and Agg4 carrier and noncarrier cases

Clinical variable	Agg4			Predis18		
	Carriers (*n* = 24)	Noncarriers (*n* = 1257)	Trend	Carriers (*n* = 87)	Noncarriers (*n* = 1194)	Trend
Age at diagnosis (yr)						
Median	58	57	*p* = 0.14 *U* = 12 470	57	57	*p* = 0.50 *U* = 54 198
Quantiles	54–59	54–58		54–58	54–58	
Range	47–60	38–60		43–60	38–60	
PSA at diagnosis (ng/ml)						
Median	29.6	8.3	*p* = 9.5 × 10^−4^ *U* = 8836	9.1	8.4	*p* = 0.57 *U* = 45 811
Quantiles	10.5–99.5	5.5–18		6–16.1	5.5–18.5	
Range	0.41–399	0.04–9020		1.1–1151	0.04–9020	
Unknown	0	43		5	38	
Gleason score (highest recorded)						
≤6	6	570		40	536	
7	2	470		35	437	
≥8	16	185		6	195	
Unknown	0	32		6	26	
Primary tumour stage at diagnosis						
T1	1	364		18	347	
T2	6	518	*p* = 1.1 × 10^−5^	40	484	*p* = 0.40
T3	9	286	*M*^2^ = 19	22	273	*M*^2^ = 0.70
T4	5	58		3	60	
T_*X*_	3	31		4	30	
Lymph node status at diagnosis						
N0	13	774		54	733	
N1	8	81	*p* = 5.6 × 10^−4^	8	81	*p* = 0.51
N_*X*_	3	402		25	380	
Distant metastases at diagnosis						
M0	18	739		52	705	
M1	4	88	*p* = 0.29	3	89	*p* = 0.26
M_*X*_	2	430		32	400	

PCa = prostate cancer; PSA = prostate-specific antigen.

Age and PSA at diagnosis, Gleason score, tumour grade, nodal spread, and metastatic statuses are shown for carrier and noncarrier PCa cases of each gene set. Tests for enrichment between carriers and noncarriers were performed for clinical variables collected at diagnosis using Mann-Whitney *U* test (age and PSA), Mantel-Haenszel test for linear-trend (tumour stage), or Fisher's exact test (nodal and metastatic spread).

## 4 Discussion

Direct sequencing approaches are required to investigate the effect of rarer germline variants in complex disease predisposition; however, to date, these studies in PCa have generally been smaller in size, considered only a handful of candidate genes, or lacked control cohorts. In this study, we investigated the role of DNA repair and damage response genes in predisposition to PCa and aggressive disease in a case/control cohort. We focused on protein truncating (tier 1) and predicted conserved (tier 2) variants using both gene-level SKAT-O and gene-set-level ADA analyses.

Gene-level analysis of tier 1 and 2 variants identified significant associations in *NBN* for PCa predisposition and *XPC* for disease aggressiveness. The *NBN* signal was refined by ADA to rs61753720, a G>T single nucleotide variant (SNV) resulting in a D95N substitution. A previous study by the ICPCG consortium found this variant at a low frequency in both unselected (1/613) and familial (1/121) Finnish PCa cohorts, and absent (0/440) in controls [26]. For the association between the *XPC* gene and a higher Gleason score, ADA selected multiple singleton SNVs across the gene. Both *POLL* and *HOXB13* were also marginally associated with PCa predisposition in the case/control analysis. Since the role of *HOXB13* rs138213197 in PCa risk has been well established, sample size may have been a limiting factor in achieving Bonferroni-corrected significance, suggesting that *POLL* may also warrant additional follow-up in larger cohorts or meta-analyses of individual studies.

Gene-set-level analysis identified 20 genes in which PTVs were associated with PCa predisposition. These included the established *BRCA1/2* genes, a handful of additional genes that have been indicated previously as prospective PCa candidates (*ATM*, *CHEK2*, *GEN1*, *MSH2*, and *RNASEL)*, and several novel genes for which limited substantive evidence for a role in PCa predisposition has been presented to date (*BLM*, *CDC25C*, *ERCC3*, *LIG4*, *MSH5*, *NEIL2*, *NHEJ1*, *PARP2*, *POLD1*, *POLE*, *POLM*, *RECQL4*, and *TDP1*). We furthermore identified four genes associated with more aggressive PCa phenotype, three of which overlapped the 20-gene PCa predisposition set. These include *BRCA2*, for which association with a more aggressive phenotype has reliably been demonstrated [6], [7], [27], [28], whilst we also present evidence that carriers of PTVs in *MSH2, CHEK2* (excluding 1100delC), and *ERCC2* also have a substantially higher likelihood of developing aggressive disease.

Our criteria to stratify cases for the aggressive phenotype analysis (Gleason score ≤7 vs ≥8) were chosen to maximise the homogeneity and risk of the aggressive group. Within the Gleason 7 category, however, Gleason 4 + 3 patients have poorer prognosis than Gleason 3 + 4 patients, with these two subgroups categorised separately according to the prognostic grade grouping method [29]. We therefore compared the results of our aggressive analysis with those of Gleason 4 + 3 cases reclassified as aggressive, equivalent to grade group ≤2 versus ≥3 (*n* = 924 vs 324) instead of grade group ≤3 versus ≥4 used for our primary analysis. Under this classification, ADA selected the Agg4 gene set alongside three additional genes (*ESR2*, *GTF2H4*, and *SETMAR*; *p*_*ADA*_ = 8.1 × 10^−3^, opt.t = 0.105). Additional comparisons between Gleason ≥8 cases and controls selects the same *Agg4* genes as our primary aggressiveness analysis (*p*_*ADA*_ = 0.014, opt.t = 0.115), whereas analysis of Gleason ≤7 cases versus controls selects 12 genes overlapping the Predis18 gene set identified in the case/control analysis (*ATM*, *BRCA1*, *CDC25C*, *CHEK2* 1100delC, *GEN1*, *LIG4*, *NEIL2*, *PARP2*, *POLD1*, *POLM*, *RECQL4*, *TDP1*; *p*_*ADA*_ = 0.029, opt.t = 0.12).

The overall 23-gene panel from the union of our gene and gene-set-level results for PCa susceptibility and disease aggressiveness spans a range of primary DNA repair pathways (Supplementary Table 1), with homologous recombination, mismatch repair, base excision repair, nucleotide excision repair, nonhomologous end joining, and DNA damage response all represented through multiple genes. Although Gleason score was used to stratify aggressive and nonaggressive disease and is correlated with other features indicative of poor prognosis, among carriers of mutations in the Agg4 gene set, we nevertheless observed substantial enrichment over noncarriers for nodal invasion (38% vs 9.5%), metastatic disease (18% vs 11%), and reduced survival (PCa-specific 5-yr survival rate 60% vs 91%), suggesting that these genes could potentially demonstrate clinical utility for the identification of individuals at a higher risk of advanced disease prior to progression. The absence of *BRCA1* and *ATM* from our aggressive gene set is however notable, as PTVs in these genes have been implicated in increased risks of metastatic and lethal PCa cancer previously [6], [7], [30]. This discrepancy may in part reflect our use of Gleason score to define aggressive disease due to the modest proportion of patients with metastatic disease in our unselected cohort (7.2% of overall cohort, 11% excluding unknown status) in comparison with the more stringent metastatic or lethality indicators employed elsewhere in cohorts enriched for these outcomes, or alternatively that these genes confer lower influence upon aggressiveness in younger patients. It is also noteworthy that whilst *CHEK2* was associated with PCa predisposition for both 1100delC and other PTVs, only the non-1100delC *CHEK2* variants were found to contribute towards aggressive disease in our study. This observation, however, contrasts with a recent report in which only the 1100delC variant and not overall *CHEK2* mutations were enriched in lethal PCa patients [31], and therefore requires further validation in independent cohorts. These combined reports could, however, potentially indicate that the downstream functional consequence of the 1100delC founder mutation may partly differ from those of other *CHEK2* PTVs in prostate tissue.

Whilst the novel genes that we have identified represent exciting candidate moderate-penetrance PCa-risk genes, these findings nonetheless require additional validation in independent cohorts. In particular, we note that the optimal *p* value truncation thresholds used by ADA are tuned towards greater sensitivity than specificity to maximise power for rare variant discovery in sequencing study sample sizes, and no suitable replication set was available for confirmation of our findings. Furthermore, even though this is the largest DNA repair gene germline sequencing study for PCa to date, our power to detect rare associations with moderate effect sizes remained modest.

Whilst our strategy of using screened controls (no PCa FH or PSA <0.5 ng/ml) potentially increased our power to detect associations, this also has the potential to introduce bias in our case/control analyses. We therefore cannot completely exclude the possibility that the use of PSA or FH in our control selection criteria led to an observed depletion of LoF variants among controls; although this would imply a uniform direction and comparatively high penetrance of effects across a wide range of DNA repair genes and pathways should these associations have been driven exclusively by extraneous variables such as low PSA levels independently of PCa.

## 5 Conclusions

In this study, we confirmed previous PCa predisposition gene reports and also present evidence for additional novel genes. Our combined gene and gene-set-level analyses provide evidence for a prospective screening panel of 23 genes that may facilitate identification of individuals at a higher PCa risk prior to disease onset, who would warrant enhanced screening. In addition, PCa patients who are carriers of mutations in these genes could potentially benefit from personalised treatment pathways [27], [32]. We believe that these genes warrant evaluation by the wider scientific and clinical communities in larger prospective studies or meta-analyses. There is also a need to formally test the ability of these genes to predict survival in an independent cohort within aggressiveness strata.

***Author contributions:*** Zsofia Kote-Jarai had full access to all the data in the study and takes responsibility for the integrity of the data and the accuracy of the data analysis.  

*Study concept and design:* Leongamornlert, Saunders, Conti, Kote-Jarai, Eeles.

*Acquisition of data:* Wakerell, Whitmore, Cieza-Borrella, Dadaev, Donovan, Hamdy, Neal, Muir.

*Analysis and interpretation of data:* Leongamornlert, Saunders, Conti, Kote-Jarai.

*Drafting of the manuscript:* Leongamornlert, Saunders, Kote-Jarai.

*Critical revision of the manuscript for important intellectual content:* Leongamornlert, Saunders, Conti, Kote-Jarai.

*Statistical analysis:* Leongamornlert, Brook.

*Obtaining funding:* Kote-Jarai, Eeles.

*Administrative, technical, or material support:* Wakerell, Whitmore, Cieza-Borrella, Benafif, Govindasami, Dadaev.

*Supervision:* Kote-Jarai, Eeles.

*Other:* None.  

***Financial disclosures:*** Zsofia Kote-Jarai certifies that all conflicts of interest, including specific financial interests and relationships and affiliations relevant to the subject matter or materials discussed in the manuscript (eg, employment/affiliation, grants or funding, consultancies, honoraria, stock ownership or options, expert testimony, royalties, or patents filed, received, or pending), are the following: None.  

***Funding/Support and role of the sponsor*****:** Funding support was provided by Cancer Research UK (grant C5047/A17528), the Prostate Cancer Research Foundation (now Prostate Cancer UK), Prostate Research Campaign UK (now Prostate Cancer UK).  

***Acknowledgements:*** We would like to acknowledge the NCRN nurses and consultants for their work in the UKGPCS study. We also wish to thank all the patients and control men who took part in this study. We also thank the Institute of Cancer Research, the Everyman Campaign, the National Cancer Research Network UK, and the National Cancer Research Institute (NCRI) UK. We are grateful for support of NIHR funding to the NIHR Biomedical Research Centre at The Institute of Cancer Research and The Royal Marsden NHS Foundation Trust.
