# Supplementary material for: Germline DNA Repair Gene Mutations in Young-onset Prostate Cancer Cases in the UK: Evidence for a More Extensive Genetic Panel
Source: Eur Urol. 2019 Sep;76(3):329–37. doi: 10.1016/j.eururo.2019.01.050 (PMC6695475; doi:10.1016/j.eururo.2019.01.050)
Supplement: Supplementary file 1 [file mmc1.pdf]

Supplementary Methods

Target design

The 175 target gene panel (Supplementary Table 1) was constructed using the following sources;

1. All 22 genes from the 22 BROCA gene panel used in Leongamornlert et al. 2014[1].

2. A DNA repair literature review compromising the DNA repair list curated by the Wood lab at MD Anderson (http://sciencepark.mdanderson.org/labs/wood/DNA_Repair_Genes.html) and another used by Kang et al. to construct a DNA repair pathway based score for ovarian cancer outcome prediction [2-6].
3. Curated DNA repair, damage response and cell cycle pathways were obtained from the following databases (accessed March 2012);

    - GeneGo (Thomson Reuters Corporation, New York, NY)
    - BioCarta (http://www.genecarta.com)
    - KEGG (http://www.genome.jp/kegg/pathway.html)
    - Reactome (http://www.reactome.org/)

    BioCarta, KEGG and Reactome data was downloaded from the Molecular Signatures Database (MSigDB) by the Gene Set Enrichment Analysis (GSEA) website [7].

Target capture and sequencing

The final target regions submitted to Agilent Technologies (Santa Clara, CA, US) consisted of 2,943 non-overlapping intervals totalling 1,480,590bp. The capture design was a SureSelectXT Custom 0.5-2.9Mb bait capture library, with 2x tiling (each of the 120bp bait overlaps neighbouring baits by 60bp). The resultant capture designed by Agilent contained 23,606 unique oligo sequences covering 1,618,074bp.

Individual libraries were prepared with NEBNext reagents modules E6050L & E6053L (New England BioLabs Inc. Ipswich, MA), each with a custom 7bp “index” from a set of 96 to allow downstream multiplexing. PCR amplification was performed with KAPA HiFi ReadyMix KK2612 (Kapa Biosystems Inc. Wilmington, MA). Libraries were quantified by PicoGreen (Life Technologies, Carlsbad, CA, US) and pooled equimolarly into batches of 48 individually indexed samples. Each pool was captured twice, with 1ug added to a single Agilent SureSelect bait library hybridisation reaction (Agilent Technologies, Santa Clara, CA, USA), thus the effective capture multiplexing level was 24x.

Two 48 samples pools with complementary barcodes were loaded per lane of an Illumina HiSeq flowcell v1.5 using an Illumina cBOT and TruSeq PE cluster kit v2 reagents (Illumina, San Diego, CA). Clustered libraries were sequenced on an Illumina HiSeq 2000 instrument using TruSeq SBS v1 reagents, to generate 2x101bp “paired end” reads.

Sequencing data analysis

The analysis pipeline is based primarily on the Genome Analysis Toolkit (GATK) using "Best Practice Variant Detection with the GATK v3" and BWA 0.5.8 is used as the sequence aligner [8, 9].

Alignment

Alignment was performed against the 1000 genomes phase 1 reference human_g1k_v37.fasta [10], using BWA 0.5.8 using the –q 5 parameter to trim low quality 3' bases (< PHRED quality 5).

Post-alignment

The raw alignment was then "cleaned" to remove mapping artefacts and recalibrate the base qualities. The GATK (v2.8-1-g932cd3a) RealignerTargetCreator and IndelRealigner modules are used to locally realign "suspicious" regions of alignment [9]. Then GATK CountCovariates was run to ascertain empirically the error rate of the per base quality scores. These scores are then used to produce a recalibrated alignment using GATK TableRecalibration.

Pre genotype QC

Samples were retained if reaching ≥80% of the target at ≥10x read depth as defined by Picard v.1.52 (http://broadinstitute.github.io/picard/) and contamination <3% as estimated by verifyBamID v1.1.1 (https://github.com/statgen/verifyBamID/releases).

Genotyping

All samples were genotyped together using the GATK (v2.8-1-g932cd3a) UnifiedGenotyper module [9]. SNV and INDELs were genotyped separately over the capture target regions to produce Variant Call Format (VCF) files [11].

Genotype QC

As the GATK Unified Genotyper is tuned for sensitivity, we implemented further genotype level filters on the VCF “FORMAT” fields, based on thresholds from Garner et al. 2011, Lim et al. 2014 and Carson et al. 2014 [12-15]. Multiallelic variants were split into their biallelic constituents using the VT function “decompose” (https://github.com/atks/vt).

1. An allele balance (AB) metric was constructed using the allelic read depth contained within the VCF AD field (allele depth; 2 integers REFdepth, ALTdepth, AB = ALTdepth/(ALTdepth+REFdepth). Genotype calls not within the following thresholds were set as missing:

    a. Homozygotes AB < 0.1 or AB > 0.9

    b. Heterozygotes AB = 0.3-0.7

2. Read depth (DP). Genotypes were set as missing if DP <8 reads (which gives a 1% chance that a truly heterozygous variant is called as reference).

3. Genotype quality (GQ). Genotypes were set as missing if GQ <20 (GQ is Phred scaled e.g. GQ = 20 equates to an error 1/100 and therefore an accuracy of 99%).

Variants were also removed if they were in low-complexity regions (LCRs). These regions were found to be enriched for artefact heterozygote genotypes in an NGS mapping study of the haploid human sample CHM1, where only homozygote calls are expected [16].

Sample QC

The per-individual association study QC procedures from Anderson et al. 2010 were applied using PLINK (1.90 beta; https://www.cog-genomics.org/plink2) and the R package "SNPRelate" [17, 18]. Due to the targeted nature of the sequencing data, discordant sex and divergent ancestry sample QC was augmented with additional genotype QC data from the same samples generated on the OncoArray SNP array platform [19]. The post genotype QC VCF was converted into PLINK 1 binary format using "--vcf-half-call missing", this removes genotypes with missing allele data due to multiallelic decomposition.

- Discordant sex information (PLINK --check-sex)
  - Samples removed if genotypic or OncoArray derived sex discordant from pro forma sex
- Outlier heterozygosity and missingness (PLINK --het/--missing) (Supplementary Figure 3)
  - Samples removed with observed heterozygosity >3 SD from the mean
  - Samples were removed with ≥10% missing data
- Relatedness (SNPRelate - snpgdsIBDMLE)
  - Duplicate samples identified using IBD (coefficients k0 & k1 both equal 0)
    - Best call rate sample per concordant duplicate pair retained
    - Removed cryptic duplicates pairs
    - Removed in plate controls
- Divergent ancestry (SNPRelate - snpgdsPCA)
  - Samples removed if outlier to 1000 genomes EUR super population
    - PCA performed on intersect of study and Phase 3 1000 genomes SNVs (Supplementary Figure 1 & 2)
    - Samples were retained if contained within an 99.99% data ellipse of study samples designated as European by OncoArray QC for both EV1/2 and EV3/4
    - Any remaining samples designated as non-European by OncoArray QC were removed

Variant level QC

After removal of samples failing any QC step, variants were reassessed using R and PLINK 1.9 and variants were removed if:

- Outside the target region as defined according to RefGene CDS for each target gene, with a 10bp flank
- The missing rate (FMISS) was greater than 10%
- There is significant ($P < 10^{-5}$) difference in missing rate between cases and controls
- The HWE test of deviation as implemented in PLINK is $P < 10^{-5}$

Supplementary Tables

Supplementary Table 1: Genes included in the 175 target gene panel grouped by primary DNA repair pathway

Consensus pathway	Gene Count	Gene List
Direct reversal repair (DRR)	3	ALKBH2, ALKBH3, MGMT
Base excision repair (BER)	25	APEX1, APEX2, APLF, DUT, LIG3, MBD4, MPG, MUTYH, NEIL1, NEIL2, NEIL3, NTHL1, NUDT1, OGG1, PARP1, PARP2, PNKP, POLB, RECQL4, SMUG1, TDG, TDP1, UNG, WRN, XRCC1
Mismatch repair (MMR)	12	MLH1, MLH3, MSH2, MSH3, MSH4, MSH5, MSH6, PCNA, PMS1, PMS2, POLD1, POLE
Nucleotide excision repair (NER)	30	CCNH, CDK7, CETN2, DDB1, DDB2, ERCC1, ERCC2, ERCC3, ERCC4, ERCC5, ERCC6, ERCC8, GTF2H1, GTF2H2, GTF2H3, GTF2H4, GTF2H5, LIG1, MMS19, MNAT1, RAD23A, RAD23B, RPA1, RPA2, RPA3, RPA4, UVSSA, XAB2, XPA, XPC
Homologous recombination (HR)	26	BARD1, BLM, BRCA1, DMC1, EME1, EME2, GEN1, HELQ, MRE11A, MUS81, NBN, RAD50, RAD51, RAD51B, RAD51D, RAD52, RAD54B, RAD54L, RBBP8, RECQL, RECQL5, SHFM1, SLX1A, SLX1B, XRCC2, XRCC3
Non-homologous end joining (NHEJ)	11	DCLRE1C, LIG4, NHEJ1, POLL, POLM, PRKDC, PRPF19, SETMAR, XRCC4, XRCC5, XRCC6
Fanconi Anaemia (FA)	19	BRCA2, BRIP1, FAAP100, FAAP24, FANCA, FANCB, FANCC, FANCD2, FANCE, FANCF, FANCG, FANCI, FANCL, FANCM, PALB2, RAD51C, SLX4, USP1, WDR48
DNA damage response (DDR)	22	ATM, ATR, ATRIP, CHEK1, CHEK2, CLK2, CLSPN, H2AFX, HUS1, MDC1, NABP2, PER1, PER2, RAD1, RAD17, RAD9A, RNF8, TOP2A, TOPBP1, TP53, TP53BP1, UBE2N
Cell cycle regulation	19	AKT1, CCNB1, CCND1, CCNE1, CDC25A, CDC25C, CDH1, CDK1, CDK2, CDK4, CDKN1A, CDKN1B, E2F1, GADD45A, MDM2, MDM4, PTEN, RB1, STK11
PCa candidates	8	AR, ESR1, ESR2, HOXB13, MSR1, NKX3-1, RNASEL, SPOP
**Total**	**175**	

Supplementary Table 2: 12 DRG study genes with < 80% bases passing coverage threshold metrics

Gene targets were assessed for the percentage passing QC thresholds (≥8 reads at base quality ≥20).

Gene	Mean GC (%)	Study target (CDS +/- 10bp flank)			
		Size (bp)	PASS (%)	Coverage Issues (%)	Mapping Issues (%)
FANCB	35.4	2740	77.2	22.8	0.0
RPA3	42.1	446	76.9	23.1	0.0
MSH4	35.8	3211	75.1	24.9	0.0
EME2	69.0	1495	71.6	28.4	0.0
PTEN	37.2	1392	68.4	16.8	14.8
RB1	37.1	3327	63.4	36.6	0.0
CCND1	61.5	988	59.0	41.0	0.0
NKX3-1	66.1	745	58.9	41.1	0.0
ERCC8	38.5	1499	54.6	45.4	0.0
SLX1A	63.8	948	7.6	0.0	92.4
GTF2H2	37.2	1516	0.0	0.0	100.0
SLX1B	63.8	948	0.0	0.0	100.0

Supplementary Table 3: Summary table of post QC variant frequencies

Summary of Tier 1 and 2 variants by MAF category (column percentages may not add up to 100% due to rounding).

MAF category	Variant class		Total
	Tier 1	Tier 2	
**Very rare** (MAF<= 0.05%)	218 (92%)	1457 (79%)	1675 (81%)
**Rare** (0.05% > MAF<= 0.50%)	15 (6.3%)	276 (15%)	291 (14%)
**Low Frequency** (0.50% > MAF<= 5.00%)	4 (1.7%)	78 (4.2%)	82 (3.9%)
**Common** (MAF> 5.00%)	1 (0.4%)	29 (1.6%)	30 (1.4%)
Total	238	1840	2078

Supplementary Table 4: Summary of samples with multiple Tier 1 mutations identified in the Predis18 and Agg4 gene panels

8 samples had germline PTVs in 2 genes within the combined panel of PCa predisposition and aggressive susceptibility candidates identified in this study.

Case Status	Age at Diagnosis	Gleason Score	T Stage	M Stage	N Stage	Died of PCa	Genes with germline PTVs
Case	53	8	T3	M1	N1	Yes	*BRCA1*, *CHEK2*
Case	43	8	T3	M_X	N_X	-	*ERCC3*, *POLE*
Case	57	7	T3	M0	N0	-	*RECQL4*, *RNASEL*
Case	53	7	T3	M0	N1	Yes	*BRCA2*, *CHEK2* (1100delC)
Case	60	6	T1	M_X	N0	Yes	*BLM*, *CHEK2* (1100delC)
Case	54	6	T2	M0	N0	-	*ATM*, *POLE*
Case	57	6	T2	M0	N0	-	*POLM*, *RECQL4*
Control	-	-	-	-	-	-	*CHEK2* (1100delC), *RNASEL*

Supplementary Table 5: Family history of prostate and other cancers among Predis18 and Agg4 carrier and non-carrier cases.

For prostate cancer, only family history among 1st degree relatives was evaluated, whilst any family history was used for all other cancers. Fisher's Exact test was used to test for enrichment.

	Predis18					Agg4				
**Cancer Site**	**Carrier Status**	**Family History No**	**Family History Yes**	**% FH**	***P* value**	**Carrier Status**	**Family History No**	**Family History Yes**	**% FH**	***P* value**
**Prostate**	non-carrier	913	231	20	0.16	non-carrier	954	250	21	0.28
	carrier	60	22	27		carrier	19	2	9.5	
**Breast**	non-carrier	930	264	22	0.69	non-carrier	981	276	22	0.082
	carrier	66	21	24		carrier	15	9	38	
**Colon**	non-carrier	974	220	18	0.32	non-carrier	1023	234	19	0.43
	carrier	67	20	23		carrier	18	6	25	
**Ovary**	non-carrier	1147	47	3.9	0.77	non-carrier	1207	50	4.0	1
	carrier	83	4	4.6		carrier	23	1	4.2	
**Bladder**	non-carrier	1136	58	4.9	1	non-carrier	1196	61	4.9	1
	carrier	83	4	4.6		carrier	23	1	4.2	
**Pancreas**	non-carrier	1150	44	3.7	0.56	non-carrier	1212	45	3.6	0.057
	carrier	83	4	4.6		carrier	21	3	13	

Supplementary Figures

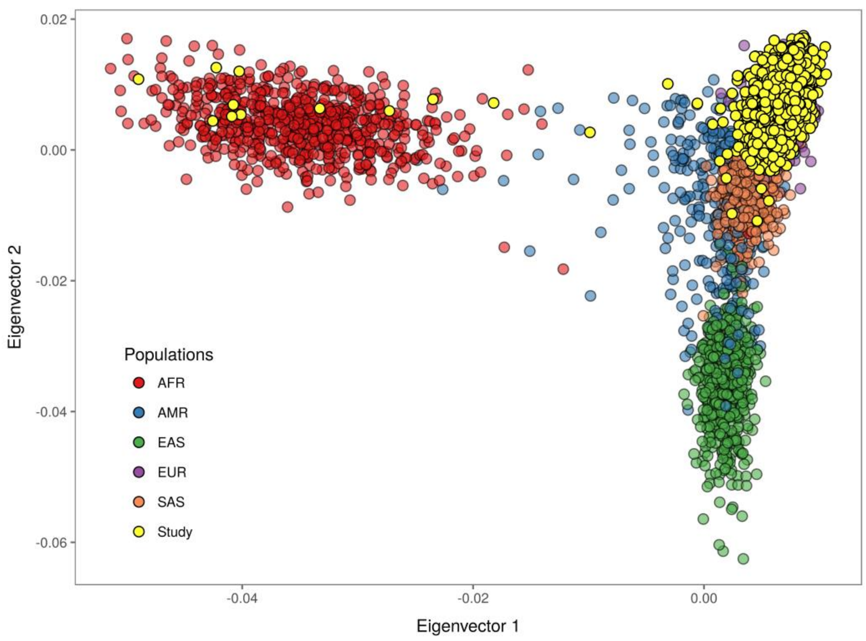

Supplementary Figure 1: EV1 and EV2 Principal Component Analysis clustering of study samples vs. 1000 Genomes Phase3

Plot of study samples (yellow) in relation to the five 1000 Genomes Project "Super populations" (AFR = African, AMR = Ad Mixed American, EAS = East Asian, EUR = European and SAS = South Asian), using eigenvectors (EV) 1 and 2.

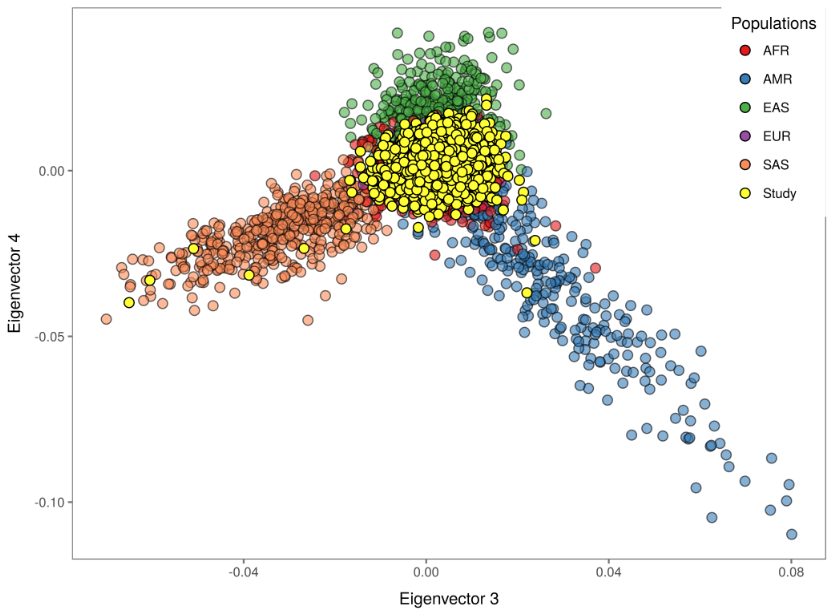

Supplementary Figure 2: EV3 and EV3 Principal Component Analysis clustering of study samples vs. 1000 genomes Phase3

Plot of study samples (yellow) in relation to the five 1000 Genomes Project "Super populations" (AFR = African, AMR = Ad Mixed American, EAS = East Asian, EUR = European and SAS = South Asian), using eigenvectors (EV) 3 and 4.

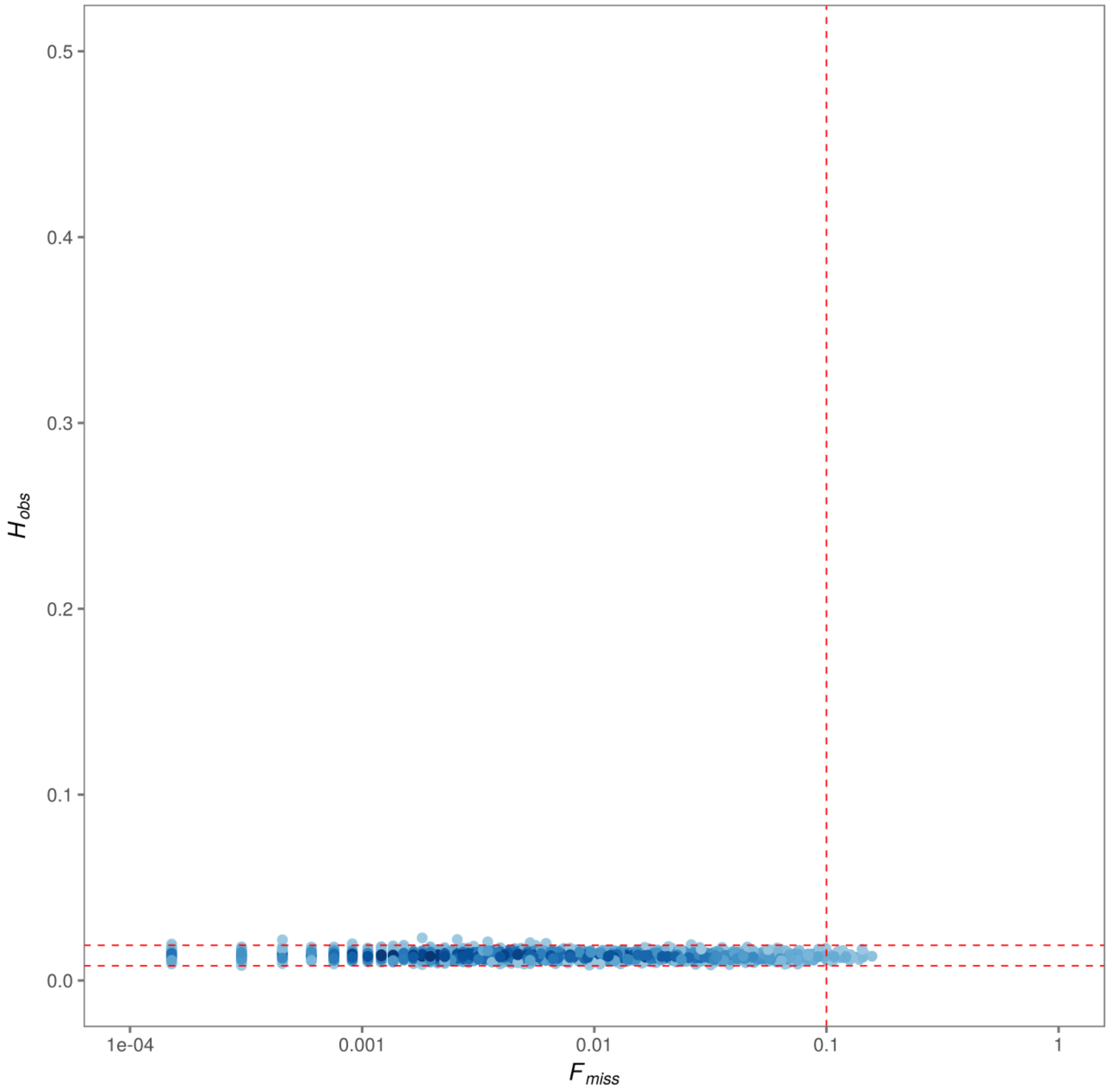

Supplementary Figure 3: Observed heterozygosity vs. Genotype missing rate

Diagnostic plot of the observed heterozygosity (H_{obs}) against genotype missingness rate (*Fmiss*) in 2568 pre-QC samples. The two horizontal red dashed lines demarcate H_{obs} +/- 3 SD from the mean and the vertical red line 10% genotype missingness.

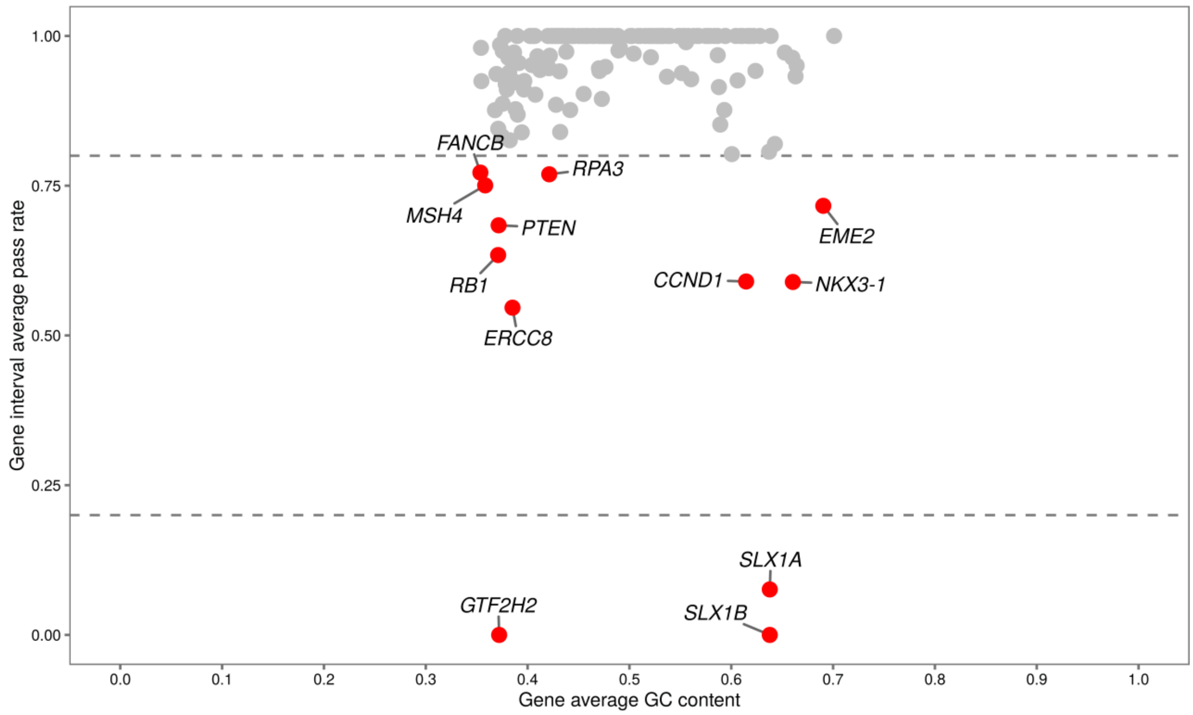

Supplementary Figure 4: Mean bases passing QC across all samples relative to average GC content per gene

Grey horizontal lines demarcate a base pair pass rate threshold (≥8 reads at base quality ≥20) of 0.8 and 0.2, 12 genes have <0.8 passing bases (highlighted in red and labelled).

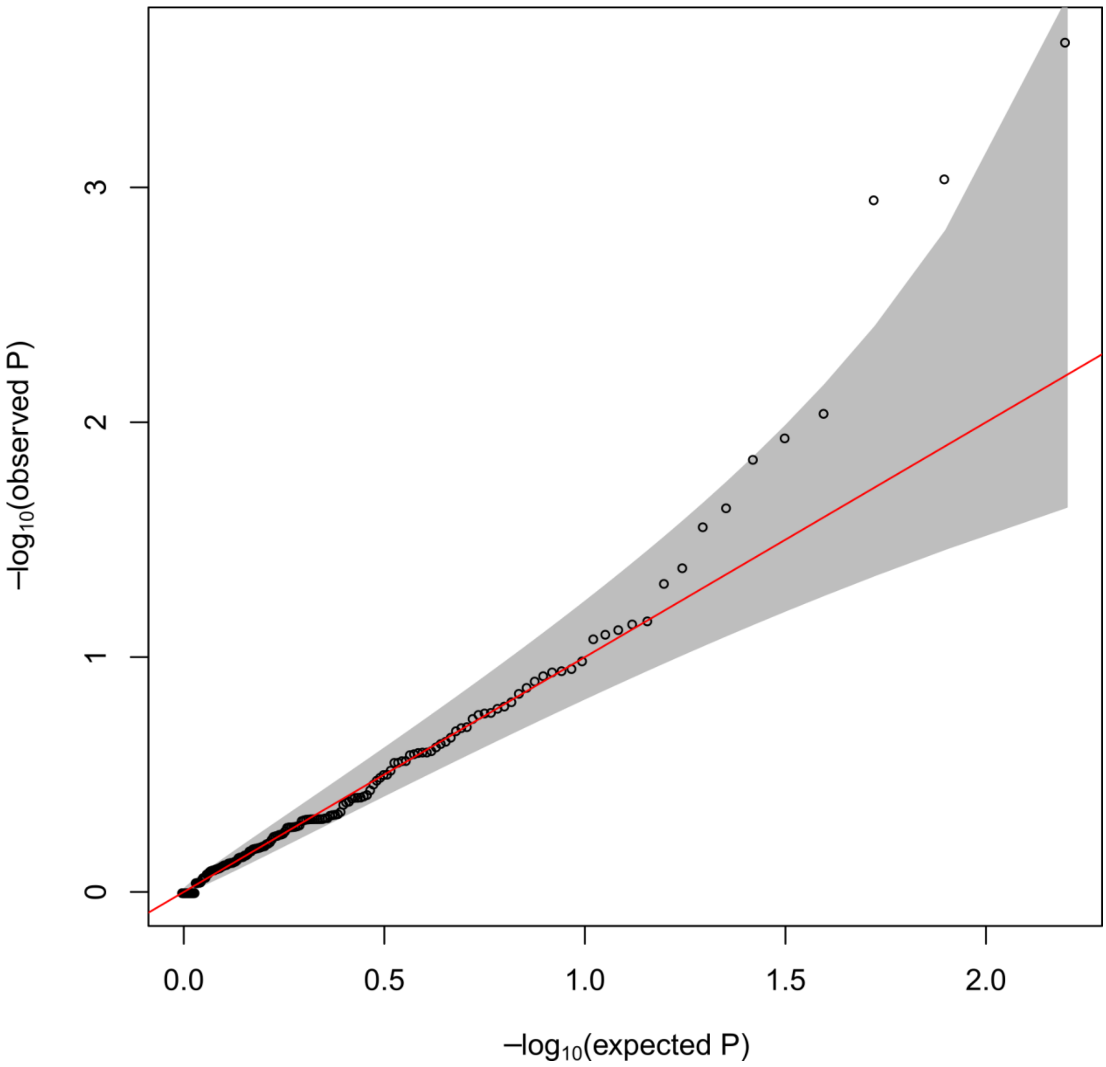

Supplementary Figure 5: Q-Q plot for case-control gene-level SKAT-O analysis

The QQ-plot shows the observed versus expected *P* values of 159 genes from 1,281 PCa cases and 1,160 controls, using the SKAT-O test. The λ = 1.03 shows slight inflation.

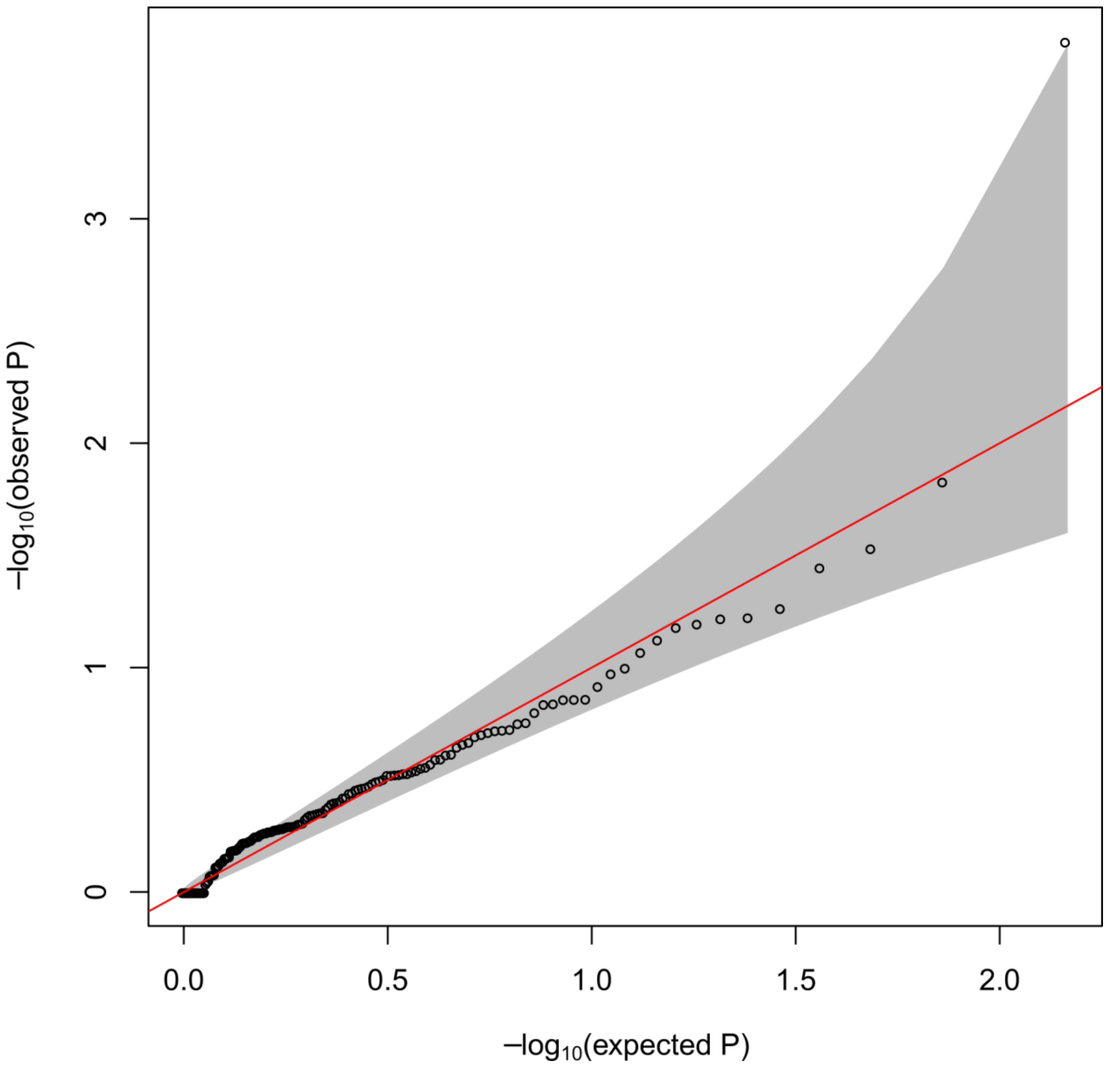

Supplementary Figure 6: Q-Q plot for aggressive phenotype gene-level SKAT-O analysis

The QQ-plot shows the observed versus expected *P* values of 146 genes from 201 aggressive (Gleason ≥8) PCa cases and 1,048 non-aggressive (Gleason ≤7) PCa cases, using the SKAT-O test. The λ = 1.12 shows slight inflation.

Gene	Non-Agg (n=1048)	Agg (n=201)	OR
RECQL4	11 (1.05%)	0 (0.00%)	0.22
ATM	7 (0.67%)	0 (0.00%)	0.34
POLM	5 (0.48%)	0 (0.00%)	0.47
RNASEL	11 (1.05%)	1 (0.50%)	0.67
CDC25C	3 (0.29%)	0 (0.00%)	0.74
NEIL2	3 (0.29%)	0 (0.00%)	0.74
TDP1	3 (0.29%)	0 (0.00%)	0.74
CHEK2_1100del	13 (1.24%)	2 (1.00%)	0.96
BLM	2 (0.19%)	0 (0.00%)	1.04
GEN1	2 (0.19%)	0 (0.00%)	1.04
PARP2	2 (0.19%)	0 (0.00%)	1.04
POLD1	2 (0.19%)	0 (0.00%)	1.04
NHEJ1	1 (0.10%)	0 (0.00%)	1.73
BRCA1	4 (0.38%)	1 (0.50%)	1.74
POLE	4 (0.38%)	1 (0.50%)	1.74
LIG4	3 (0.29%)	1 (0.50%)	2.23
MSH5	3 (0.29%)	1 (0.50%)	2.23
BRCA2	7 (0.67%)	6 (2.99%)	4.62
ERCC3	1 (0.10%)	1 (0.50%)	5.22
MSH2	1 (0.10%)	2 (1.00%)	8.75
ERCC2	0 (0.00%)	2 (1.00%)	26.28
CHEK2_non1100del	0 (0.00%)	6 (2.99%)	69.72

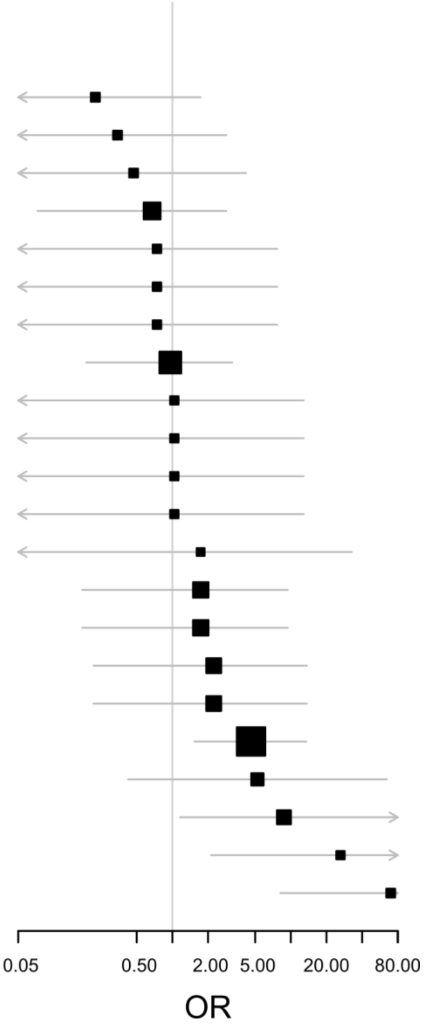

Supplementary Figure 7: Forest plot of aggressive phenotype odds ratios on the combined Predis18 and Agg4 gene sets

Supplementary References

[1] Leongamornlert D, Saunders E, Dadaev T, Tymrakiewicz M, Goh C, Jugurnauth-Little S, et al. Frequent germline deleterious mutations in DNA repair genes in familial prostate cancer cases are associated with advanced disease. Br J Cancer. 2014;110:1663-72.

[2] Friedberg EC, Walker GC, Siede W, Wood RD, Schultz RA, Ellenberger T. DNA Repair and Mutagenesis, Second Edition: American Society of Microbiology; 2006.

[3] Kang J, D'Andrea AD, Kozono D. A DNA repair pathway-focused score for prediction of outcomes in ovarian cancer treated with platinum-based chemotherapy. J Natl Cancer Inst. 2012;104:670-81.

[4] Lange SS, Takata K, Wood RD. DNA polymerases and cancer. Nat Rev Cancer. 2011;11:96-110.

[5] Wood RD, Mitchell M, Lindahl T. Human DNA repair genes, 2005. Mutat Res. 2005;577:275-83.

[6] Wood RD, Mitchell M, Sgouros J, Lindahl T. Human DNA repair genes. Science. 2001;291:1284-9.

[7] Subramanian A, Tamayo P, Mootha VK, Mukherjee S, Ebert BL, Gillette MA, et al. Gene set enrichment analysis: a knowledge-based approach for interpreting genome-wide expression profiles. Proc Natl Acad Sci U S A. 2005;102:15545-50.

[8] Li H, Durbin R. Fast and accurate short read alignment with Burrows-Wheeler transform. Bioinformatics. 2009;25:1754-60.

[9] McKenna A, Hanna M, Banks E, Sivachenko A, Cibulskis K, Kernytsky A, et al. The Genome Analysis Toolkit: a MapReduce framework for analyzing next-generation DNA sequencing data. Genome Res. 2010;20:1297-303.

[10] The 1000 Genomes Project Consortium. A map of human genome variation from population-scale sequencing. Nature. 2010;467:1061-73.

[11] Danecek P, Auton A, Abecasis G, Albers CA, Banks E, DePristo MA, et al. The variant call format and VCFtools. Bioinformatics. 2011;27:2156-8.

[12] Carson AR, Smith EN, Matsui H, Braekkan SK, Jepsen K, Hansen JB, et al. Effective filtering strategies to improve data quality from population-based whole exome sequencing studies. BMC Bioinformatics. 2014;15:125.

[13] Chan Y, Lim ET, Sandholm N, Wang SR, McKnight AJ, Ripke S, et al. An excess of risk-increasing low-frequency variants can be a signal of polygenic inheritance in complex diseases. Am J Hum Genet. 2014;94:437-52.

[14] Garner C. Confounded by sequencing depth in association studies of rare alleles. Genet Epidemiol. 2011;35:261-8.

[15] Lim ET, Wurtz P, Havulinna AS, Palta P, Tukiainen T, Rehnstrom K, et al. Distribution and medical impact of loss-of-function variants in the Finnish founder population. PLoS Genet. 2014;10:e1004494.

[16] Li H. Toward better understanding of artifacts in variant calling from high-coverage samples. Bioinformatics. 2014;30:2843-51.

[17] Anderson CA, Pettersson FH, Clarke GM, Cardon LR, Morris AP, Zondervan KT. Data quality control in genetic case-control association studies. Nat Protoc. 2010;5:1564-73.

[18] Zheng X, Levine D, Shen J, Gogarten SM, Laurie C, Weir BS. A high-performance computing toolset for relatedness and principal component analysis of SNP data. Bioinformatics. 2012;28:3326-8.

[19] Schumacher FR, Al Olama AA, Berndt SI, Benlloch S, Ahmed M, Saunders EJ, et al. Association analyses of more than 140,000 men identify 63 new prostate cancer susceptibility loci. Nat Genet. 2018;50:928-36.
